# IGFBP-2 and IGF-II: Key Components of the Neural Stem Cell Niche? Implications for Glioblastoma Pathogenesis

**DOI:** 10.3390/ijms26104749

**Published:** 2025-05-15

**Authors:** Abigail J. Harland, Claire M. Perks

**Affiliations:** Cancer Endocrinology Group, Bristol Medical School, Translational Health Sciences, University of Bristol, Southmead Hospital, Bristol BS10 5NB, UK; ah15974@bristol.ac.uk

**Keywords:** neural stem cells, central nervous system, subventricular zone, hippocampus, cerebral spinal fluid, glioblastoma, glioma stem cells

## Abstract

Glioblastoma is a fatal and aggressive cancer with no cure. It is becoming increasingly clear that glioblastoma initiation is a result of adult neural stem cell (NSC) transformation—most likely those within the subventricular zone (SVZ). Indeed, transcriptomic analysis indicates that glioblastomas are reminiscent of a neurodevelopmental hierarchy, in which neural stem and progenitor markers are widely expressed by tumour stem-like cells. However, NSC fates and the cues that drive them are poorly understood. Studying the crosstalk within NSC niches may better inform our understanding of glioblastoma initiation and development. Insulin-like growth factor binding protein 2 (IGFBP-2) has a well-established prognostic role in glioblastoma, and cell-based mechanistic studies show the independent activation of downstream oncogenic pathways. However, IGFBP-2 is more commonly recognised as a modulator of insulin-like growth factors (IGFs) for receptor tyrosine kinase signal propagation or attenuation. In the adult human brain, both IGFBP-2 and IGF-II expression are retained in the choroid plexus (ChP) and secreted into the cerebral spinal fluid (CSF). Moreover, secretion by closely associated cells and NSCs themselves position IGFBP-2 and IGF-II as interesting factors within the NSC niche. In this review, we will highlight the experimental findings that show IGFBP-2 and IGF-II influence NSC behaviour. Moreover, we will link this to glioblastoma biology and demonstrate the requirement for further analysis of these factors in glioma stem cells (GSCs).

## 1. Introduction

Glioblastoma is a fatal brain cancer with limited therapeutic options and a survival time of 15–18 months [1,2]. The most common diagnostic age range is 55–85 years, with an incidence of approximately 3% in children [3]. Earlier intervention in disease pathology is hoped to lead to novel treatment approaches and better patient outcomes [4]. Of note, the overexpression of insulin-like growth factor binding protein 2 (IGFBP-2) in glioblastoma tumour tissue has been repeatedly reported and is associated with shorter patient survival [5,6,7,8,9]. In efforts to rationalise this prognostic effect, in vitro glioblastoma studies suggest that IGFBP-2 frequently functions independently of insulin-like growth factor (IGF) signalling [10,11,12,13]. Instead, functional motifs within the structure are thought to promote the survival, expansion and migration of immortalised glioblastoma cell lines [14]. However, glioblastoma is a poorly differentiated tumour. Progression is frequently compared to the processes that take place during pre- and postnatal neurodevelopment. Thus, there is good evidence to suggest that glioblastoma is a stem cell-driven cancer, propagated from a non-malignant neural stem cell origin [15]. Therefore, further to previously delineated observations of *IGFBP2* overexpression in tumour tissue and immortalised cell lines, emerging evidence suggests additional roles for the maintenance of glioblastoma cells with stem cell properties—commonly referred to as glioma stem cells (GSCs) [16]. However, IGF-II must not be ignored. It is becoming increasingly clear that *IGF2* expression surrounding neurogenic niches in the adult human brain (the subventricular zone (SVZ) or the subgranular zone (SGZ)) can influence cell fate [17]. *IGF2* is an imprinted gene and therefore the paternal allele is expressed by most tissues, as well as the SGZ [18]. However, biallelic expression is present in the SVZ neurogenic niche [18]. The delineation of these differences has been related to contrasting functionalities, such that IGF-II can act in a paracrine manner as a mitogen in the SVZ, or in an autocrine manner in the SGZ as a survival factor [17]. Indeed, these findings could have implications for delineating the mechanisms that take place during glioblastoma development.

This review will focus on the functions of IGFBP-2 in the maintenance of neural stem cells (NSCs) and their malignant counterparts (GSCs). Due to the overexpression of *IGFBP2* within tumour tissue, many studies focus on the functions of IGFBP-2 in glioblastoma. However, whilst the study of IGFBP-2 is more robust, IGF-II secretion into the cerebral spinal fluid (CSF) similarly positions it near key neurogenic regions. Therefore, despite a lack of study in GSCs, evidence from NSC experiments highlights IGF-II as a biologically plausible yet underexamined contributor to the glioblastoma microenvironment.

## 2. IGF and Insulin Axis Components

**Figure 1:**
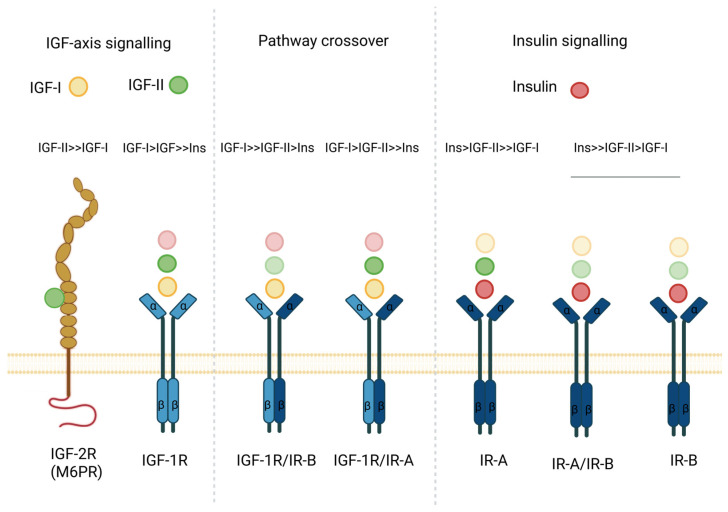
The insulin-like growth factor (IGF)/insulin axis: receptors and ligands. Receptors include transmembrane receptors (the IGF-1 receptor (IGF-1R) and the insulin receptor (IR)), hybrid heterodimers (IR-A and IR-B) and hybrid tetramers (IGF-1R/IR). Hybrid receptors are formed from the heterodimerisation of IGF-1R and IR subunits. Ligands include insulin, IGF-I, IGF-II and their variants (high-molecular-weight IGF-II). IGF-2R/mannose 6-phosphate receptor (M6PR) is a transmembrane glycoprotein without kinase activity. Relative binding affinities for each receptor–ligand interaction differ. Insulin binding affinity is strongest for the IR-A and IR-B homodimers and weaker for heterodimeric receptors. IGF-I binding affinity is strongest for the IGF-1R, IGF-1R/IR-A and IGF-1R/IR-B heterodimers. IGF-II binding affinity is strongest for the IGF-1R, IR-A and IGF/IR-A heterodimeric receptor. Overarching biological effects for receptor activation via these pathways include the modulation of metabolism and mitogenesis. Figure adapted from [27,28,29,30]. Created with BioRender.com.

IGF and insulin signalling coordinate several biological processes with overarching outcomes including the modulation of metabolism and mitogenesis. The IGF/insulin signalling axis comprises ligands: insulin, IGF-I, IGF-II with variants (high-molecular-weight IGF-II), two structurally homologous transmembrane receptor tyrosine kinases (the IGF-I receptor (IGF-1R) and the insulin receptor (IR)), along with hybrid heterodimers (IR-A and IR-B) and hybrid tetramers (IGF-1R/IR, (Figure 1). IGF-2R, also known as the mannose-6-phosphate (M6P) receptor, is a type I transmembrane protein, lacks kinase activity and is structurally distinct from IR and IGF-1R [19]. IGF-2R binds to several ligands including those containing mannose-6-phospate to mediate lysosomal enzyme trafficking and degradation, as well as IGF-II, resulting in signal attenuation and growth modulation [20,21]. The IGF system additionally includes IGFs distributed between the cell surface (IGF-II/the mannose-6-phosphate (M6P) receptor) and secreted into the extracellular environment. Six IGF-binding proteins (IGFBP-1 to 6) rapidly bind IGFs, increasing their half-life and relative bioavailability [22,23,24,25]. Notably, and in the context of the current review, the binding affinity between IGFBP-2 and IGF-II is 10–20-fold greater than that of IGF-I [26].

For an extensive review of IGF and insulin signalling in physiology and disease, please refer to the review by LeRoith et al. (2021) [27].

The early identification and description of IGFBPs was as IGF carrier proteins without independent biological activity [31]. By 1991, each of the six IGFBPs had been identified and discriminated via differing molecular weights. Sources included human serum, CSF, rat serum and conditioned media (CM) from the culture of human cells [32,33,34,35,36,37,38,39]. IGFBP-1, 2, 4 and 6 bind IGFs in a binary fashion, forming complexes of 30–40 kDa and increasing their half-life from 10 to 30–90 min [24]. Greater half-life extension to 12–20 h is achieved by ternary complexing of IGFs with IGFBP-3/5 and the acid-liable subunit [24,40,41,42]. The binding affinity of IGFBPs to IGF-I and IGF-II is greater than between the ligands and receptors, thus modulating ligand–receptor binding and activation [43]. IGFBPs provide a further layer of sophistication to the IGF axis, due to spatial and temporal tissue expression, as well as IGF binding affinity. For IGF release, IGFBPs undergo fragmentation via extracellular proteases with different specificities such as pregnancy-associated plasma protein-A (PAPP-A) or matrix metalloproteinase 9 (MMP-9), which target sites within the central linker domain of IGFBP-2, 4 and 5 [44,45,46,47]. Further subtle modulation of IGF activity can be achieved through high-affinity extracellular matrix (ECM)-IGFBP binding which can either reduce or potentiate IGF signalling [48].

## 3. IGFBP-2, IGF-II and the Adult Human Brain

In the 1990s, immunohistochemistry (IHC) and in situ hybridisation (ISH) studies sought to delineate the roles of IGFs throughout embryogenesis, with many reporting distinct distribution patterns [49,50]. Of particular interest was the developing central nervous system (CNS), which is tightly regulated by growth factors including IGFs [51,52]. For example, widespread embryonic expression of *IGF1* can be detected in neurones and glial cells, with region-specific expressions including the olfactory bulb [50,53,54,55,56,57,58]. Postnatally, peak *IGF1* expression coincides with rapid neurogenesis, neuronal proliferation and myelination in the second week after birth, with the highest detectable levels in the olfactory bulb and hippocampus [54,59,60,61]. *IGF2* on the other hand is expressed at its highest levels prenatally with widespread messenger ribonucleic acid (mRNA) expression including the choroid plexus (ChP), leptomeninges, hypothalamus and floor of the third ventricle [53,62]. In adults, IGF-II is retained at higher levels than IGF-I, with biallelic expression in the meninges, ChP and vascular endothelial cells [18,63,64,65]. *IGF2* can also be monoallelically expressed by SGZ NSCs [17,18]. The differences between biallelic expression in the SVZ niche and monoallelic expression in the SGZ appear to be of functional importance, with mitogenic paracrine outcomes in the SVZ, and cell survival autocrine outcomes in the SGZ [18]. Moreover, a deficiency in *IGF2* in vivo appears to impair SVZ NSC proliferation [18]. In addition to mature IGF-II, higher-molecular-weight variants which are stable but have undergone incomplete processing of the prohormone have been detected in adult human serum [66]. It is worth noting that high-molecular-weight variants predominate in several malignancies and are considered the principal circulating form in tumour-associated hypoglycaemia, as reviewed by Scalia et al. (2023) [67]. In 1985, Haselbacher et al. identified several molecular weight forms of IGF-II including 13 kDa, 26 kDa and 38 kDa in addition to mature 7.5 kDa IGF-II across 24 regions of the human brain, and high-molecular-weight IGF-II had previously been detected in human spinal fluid [68,69]. Despite the 9 kDa variant in CSF exhibiting biological activity via radioimmunoassay, the effects on the human brain are not yet fully understood [68].

The regulatory pathways underlying the control of IGF component levels in the brain for normal homeostasis are relatively understudied. Current knowledge of adult IGF induction is mainly derived from brain injury and pathology [70,71]. Spatial and temporal expression and abundance of IGFBPs have also been closely studied to determine the neuroanatomical distribution and synchronous effects on IGF-mediated CNS signalling [71,72,73,74]. In the adult human brain, IGFBP-2 is the most abundant IGFBP and is secreted into the CSF from ChP [75,76]. Moreover, functional IGFBP-2 produced in the brain is distinct to that of circulating plasma following the establishment of the CSF–blood barrier [76,77]. IGF-II expression is also retained in the ChP, and free IGFs can be actively transported across the blood–brain barrier (BBB) into adulthood [75,78].

### 3.1. Neural Stem Cells Are Retained in the Adult Human Brain

**Figure 2:**
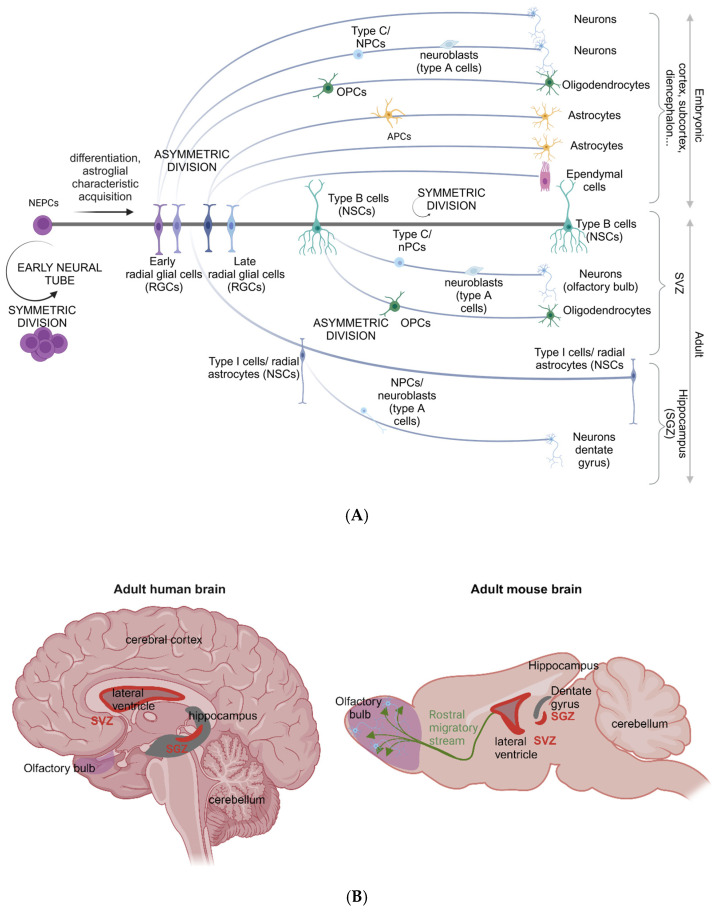
Neural stem cell lineage tree and location in human and mice. (**A**) Neuroepithelial cells (NEPCs) arise during neural tube formation and can undergo symmetric division, supporting development and retaining their population. The differentiation of NEPCs and adoption of astroglial characteristics give rise to early and late radial glial cells (RGCs). RGCs undergo asymmetric division, giving rise to neural stem cels (NSCs) (type B cells in the subventricular zone (SVZ) or type I cells in the subgranular zone (SGZ)) which persist postnatally/into adulthood and have the potential to form differentiated progenies. NSC differentiation gives rise to multipotent progenitor cells in the SVZ (type C) and SGZ (type II cells). Downstream differentiation into neural progenitor cells (NPCs)/type C cells, oligodendrocyte progenitor cells (OPCs) and astrocyte progenitor cells (APCs) has the capacity to form progenies of neurones, oligodendrocytes, astrocytes and ependymal cells throughout embryonic brain development. OPCs can stay undifferentiated and continue to proliferate in the adult brain. NSCs can also directly differentiate into mature cell types. NSCs in the SVZ generate type C cells which can form type A cells. Type A cells migrate into the olfactory bulb for neurogenesis, whereas SGZ NSCs generate neurones locally in the dentate gyrus [86]. Adapted from [100]. (**B**) Schematic showing the location of adult NSCs in the SVZ and SGZs of human and mice brains. Adapted from [101,102]. Created with BioRender.com.

Cells of the CNS that maintain neurogenesis from embryogenesis into adulthood in mammals were first reported in the 1960s using thymidine-H^3^ autoradiographic labelling in rat and cat brains [79,80]. Adult NSCs are believed to arise from embryonic radial glial cells (RGCs) via transient foetal brain structures such as the ganglionic eminences [81,82,83,84]. Bromodeoxyuridine labelling of SGZ and SVZ human brain tissue postmortem by Eriksson et al. (1998) showed that progenitor cells resided in both these regions and were capable of neurogenesis [85]. Further analysis of NSCs from each region found that NSCs in the SVZ (or type B cells) were reminiscent of astroglia, expressing markers of self-renewal (sex determining region Y-box 2 (SOX2) and nestin), markers associated with the astrocyte lineage (glial fibrillary acidic protein (GFAP), glutamate aspartate transporter, tailless and vascular cell adhesion molecule 1 [86,87,88]. NSCs of the SVZ have been shown to give rise to neurones and oligodendrocytes [86,89]. On the other hand, NSCs identified in the SGZ (radial astrocytes/type I cells), defined by SOX2, Nestin and GFAP expression, could self-renew and mainly function as precursors for postnatal neurogenesis [90,91,92,93] **(**Figure 2A).

Much of the evidence for NSC development in the mammalian brain has been derived from rodent studies [94]. Type I cells in the SGZ have the capacity to generate neurones and dentate-granule cells, contributing to spatial learning and short-term memory [95,96]. However, in the SVZ, NSCs form type C multipotent progenitors, which in turn can form neuroblasts (type A cells) that can migrate to the olfactory bulb along the rostral migratory stream to generate interneurons [86,94]. In humans, the SVZ is a 3–5 mm thick region that also contains NSCs [97]. However, in contrast to rodents, there is no evidence for the rostral migratory stream [98]. Moreover, olfactory-bulb-localised interneuron production has been detected in human infants up to the age of 18 months [99] (Figure 2B).

### 3.2. The Neural Stem Cell Secretome: IGFBP-2 and IGF-II

The NSC niche is a term used to describe the specialised microenvironment that supports cell behaviours: self-renewal, proliferation and differentiation. For example, cells that may directly influence NSCs through cellular contact, autocrine/paracrine communication include NSCs themselves, astrocytes, ependymal cells, type C cells, ChP epithelial cells, vascular endothelial cells and microglia [18,103,104].

NSC secreted factors or extracellular vesicles could result in autocrine and/or paracrine regulation of other NSCs or cell types in the brain with diverse biological effects depending on the molecular features of the recipient cell [105,106,107]. As reviewed by Dause et al. (2022), the secretome of NSCs varies across neurodevelopment [105]. For example, secreted molecules such as sonic hedgehog (Shh) are required for the proliferation and differentiation of RGCs in the ventricular zone after neural tube formation [108]. However, NSCs in adults require Shh signalling for self-renewal and maintenance [109]. Thus, molecules such as these are spatially and temporally regulated for tightly controlled localised signalling required at different developmental stages.

Historically, trophic factors, chemokines and cytokines have been measured using pre-selected antibody arrays or liquid chromatography–tandem mass spectrometry (LC-MS/MS) of CM produced by cells grown in serum-free conditions [110,111]. In a study by Cervenka et al. (2021), proteomic analysis of the CM collected from NSCs (derived from human embryonic stem cells (ESCs)) was compared with those subjected to growth factor withdrawal for different durations [110]. The group found that subject to differentiation, NSC secreted protein profiles undergo significant global changes. Notably, following 21 days of epidermal growth factor (EGF) and fibroblast growth factor (FGF2) withdrawal, IGFBP-2 showed a −2.6-fold change relative to day 0, supporting the notion that IGFBP-2 abundance is decreased upon neuronal differentiation [110]. IGF-II was not detected in this study. Previously, the inclusion of IGFBP-2 in pre-selected antibody arrays has led to detection in CM from NPCs (derived from human ESCs) [111]. In contrast, other analyses of neurosphere-derived CM using mass spectrometry have not detected IGFBP-2 or IGF-II. Instead, molecules such as apolipoprotein E (APOE), DSD-1 proteoglycan and chondroitin sulfate proteoglycan were detected, required for stimulating neurosphere formation and proliferation in vitro [112].

Improved approaches have recently emerged, integrating both in vivo and in vitro assessment to provide a more comprehensive picture of secretomes in both contexts [113]. Lee et al. (2012) created secretory molecule expression profiles from isolated cells: SVZ NSCs, type C cells, ependymal cells, astrocytes and vascular cells [103]. Notably, in this study, previously identified *APOE* was highly expressed by all niche cells analysed. However, *IGFBP2* showed specific expression in ChP cells. Surprisingly, given its expression in the ChP, *IGF2* was not reported in any of these cell types in this study [103]. Other approaches using protein arrays and LC-MS/MS have been used to characterise novel factors and facilitate comparison between transcript/protein abundance. Using these approaches to analyse mouse-derived hippocampal NSCs, Denninger (2020) revealed many proteins that have previously remained undetected [114]. Notably, three IGF binding proteins (IGFBP-2, IGFBP-3 and IGFBP-5) showed high signal intensity in antibody arrays and unbiased LC-MS/MS. Further enzyme-linked immunosorbent assays of NPSC CM and lysates showed particularly high concentrations of IGFBP-2 (10-fold greater than vascular endothelial growth factor (VEGF) which is a key protein for NPSC maintenance) [115,116]. Surprisingly, despite the high expression of *IGF2* previously detected in hippocampal NSCs, IGF-II was not detected. Despite these improved approaches, technical limitations remain in studying the NSC niche. Notably, many of the bioinformatics tools employed are still limited by profile size and may still not capture many of the essential factors required for NSC identity [103].

### 3.3. The SVZ Niche, IGFBP-2 and IGF-II

**Figure 3:**
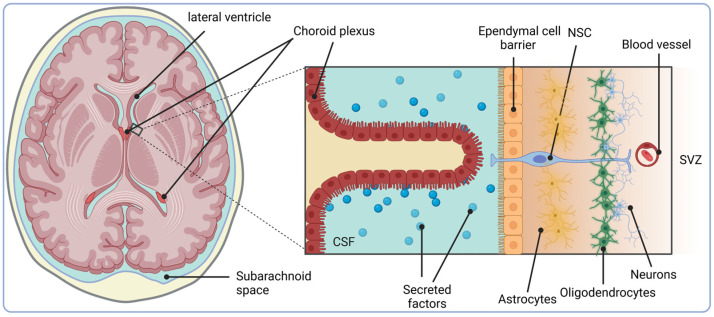
Architecture and location of the choroid plexus (ChP) and SVZ. The cellular architecture of the lateral ventricle ChP is shown. The human SVZ is composed of four layers: (1) the ependymal cell layer, lining the lateral ventricle and separating the cerebral spinal fluid (CSF) from the SVZ; (2) the hypocellular layers composed of astrocytes and microglia; (3) the additional layer formed form three different types of astrocyte; (4) oligodendrocytes and myelinated axons. NSCs contact the CSF via apical cilia at the ventricular surface. The CSF and diffusible factors are released by the ChP which can intricately mediate NSC development. Adapted from [126,127]. Created with BioRender.com.

In the adult human brain, one of the major sites for the retention of IGF-II and IGFBP-2 abundance is the ChP [76]. The ChP is a tissue present in the third, fourth and lateral ventricles of the brain from which CSF is secreted [117,118]. This structure is highly vascularised and constitutes the blood–CSF barrier to restrict solute flow via epithelial tight junctions [117] (Figure 3). CSF provides a rich source of mitogens and growth factors to the brain with a unique biochemistry to that of plasma [119]. Due to the close spatial proximity of the SVZ to the ChP, adult SVZ NSC processes can contact the CSF via apical primary cilia [120,121,122] (Figure 3). Indeed, it has been shown that pinwheel structures are set up in which ependymal cells surround apical cilia of type B NSCs [121]. Therefore, the CSF must be considered as an extremely important component of the NSC niche. Notably, CSF blockage or composition changes inhibit the proliferation of neural precursors, inhibit neuronal migration and result in abnormal cortex development [123,124,125].

Many early studies sought to delineate the complexity of the CSF proteome in rats and humans [120,128,129,130,131]. However, the first to show that the fate decisions of neural progenitors could be mediated by CSF-derived IGFs was published in 2011 by Lehtinen et al. [120]. The authors described intricate mechanisms by which protein associated with lin 7 (Pals1) and phosphatase and tensin homolog (PTEN) spatially regulate the localisation of the IGF-1R to the SVZ NSC apical complex in mice, thus governing the transduction of signals from the CSF [120]. Other examples of essential cell polarity proteins/mediators of extrinsic IGF-I/insulin signalling are α-E-catenin in skin keratinocytes and β-catenin in oligodendrocyte precursor cells (OPCs). Furthermore, IGF-II was identified as the predominant CSF-derived IGF, with 10.7-fold higher mRNA expression in adult rat ChP tissue than the cortex, potentiating NSC IGF-1Rβ, Akt, mitogen-activated protein kinase (MAPK) phosphorylation and neurosphere maintenance in vitro. Notably, CSF derived from IGF-II null mice was insufficient to stimulate the proliferation of cortical progenitors, resulting in an overall reduction in brain size (24%) [120]. Further CSF screening revealed the presence of well-established proteins involved in morphological patterning and neurogenesis including Wnts, bone morphogenic proteins (BMPs), transforming growth factor (TGF)-β factors and retinoic acid [120,132,133,134,135].

In 2015, Lun et al. investigated regional differences between choroid plexi and derived CSF from the lateral and fourth ventricles [136]. Ribonucleic acid (RNA)-sequencing analysis of bulk ChP tissue demonstrated vast region specificity with 684 differentially expressed genes across the two sites. At E18.5, *IGF2* was upregulated in the fourth ventricle ChP and *IGFBP2* was expressed in both [120,136,137]. Furthermore, IGF-II and IGFBP-2 were in the top 25 abundantly secreted proteins from ChP epithelial tissue [136]. An investigation of adult primate and human ChP samples also showed a high abundance of Shh and IGF-II [136]. Finally, in 2016, independent transcriptomic analysis of ChP tissue and the corresponding secretome in adult mice further identified growth factors, hormones and ECM remodelling proteins including FGF2, VEGF-A, IGF-I, -II, IGFBP-2,-3, SERPINE1, tissue inhibitor matrix metalloproteinase 1 (TIMP1) and MMP-8 [122]. The composition of factors was dynamically regulated with age [122]. Unsurprisingly, given essential prenatal neurogenic effects, IGF-I was one of the most enriched factors in the ChP secretome of 2-month-old mice [122]. However, a 14% decline in IGF-I has been shown to occur per decade [138,139]. Furthermore, the levels of IGF-II also reduce with age [140]. The group concluded that intricate combinations of secreted factors change over time and may reflect dynamic NSC/progenitor fates [122].

Further studies to analyse the effects of IGF-II on NSC fates have corroborated other findings in which IGF-II facilitates NSC expansion and self-renewal [17,120,122,139]. In NSCs isolated from the hippocampus dentate gyrus, *IGF2* appears to be highly expressed and can influence neurogenesis in an autocrine manner [17]. In contrast, extremely low measurements of *IGF2* in SVZ-derived NSCs confirm that it is not endogenously expressed in these cells [17]. These results corroborate the fact that IGF-II appears to function in a paracrine manner in the SVZ niche—secreted from the ChP epithelium and available to NSCs via the CSF [17,120,139]. IGF-II is also secreted from the leptomeninges and vasculature of the brain [17,18]. In contrast to studies in hippocampal NSCs—in which IGF-II was mainly shown to activate IGF-1R to regulate proliferation, by knocking out IGF-1R, Ziegler et al. (2012) showed that IGF-II can promote stemness of neural stem/progenitor cells (NSPCs) through the activation of IR-A—distinct to the actions of IGF-I [17,139]. A comparison of outcomes from IGF-I and IGF-II treatment revealed that NPSCs treated with IGF-II had increased expansion and increased mRNA expression of *OCT4*, *SOX1* and *fatty acid binding protein 7* (*FABP7*) [139]. By comparison, it is the interaction between IGF-II and other imprinted genes that has more recently been identified as a major influence for differentiation and lineage specification [141]. Lozano-Urena et al. (2023) showed that adult NSCs treated with recombinant IGF-II showed reduced stemness, and under differentiation conditions (mitogen withdrawal and addition of 2% foetal bovine serum (FBS)) promoted terminal differentiation [141]. Mechanistic analysis revealed that IGF-II activation of the phosphatidylinositol 3-kinase (PI3K)/Akt pathway mediates expression of the maternal *Cdkn1c* allele, inducing terminal differentiation under these conditions. Therefore, the retention and maintenance of the NSC pool may be reliant upon the interaction between IGF-II and *Cdkn1c* [141].

Despite a significant decrease by the third week after birth, IGFBP-2 levels remain the highest of all IGFBPs in the human CNS, particularly in the ChP and meninges [75]. However, thus far there are just two publications that study the effects of recombinant IGFBP-2 on NSCs. In the study by Wang et al. (2017), the treatment of a mouse NSC line with recombinant IGFBP-2 enhanced in vitro proliferation [142]. Moreover, the group claimed that IGFBP-2 treatment inhibited differentiation to the neuronal lineage but enhanced astrocytic differentiation [142]. Despite these findings, the culture of NSCs in this study included the use of 10% FBS—a differentiation enhancer which is typically omitted from stem cell culture systems [142]. In 2023, studies by Shahin et al. showed that IGFBP-2 can be detected in the CM of adult mouse-derived SVZ NSCs using LC-MS/MS. In this study, they proposed that the effects of self-secreted IGFBP-2 on SVZ NSCs are redox-dependent [143]. NSC proliferation and differentiation have previously been shown in response to reactive oxygen species (ROS); however, it appears that redox-sensitive proteins may mediate specific effects [144,145]. Therefore, the presence and amount of ROS present in the microenvironment may be vital for influencing the balance between self-renewal and differentiation. The increased abundance of ROS was shown to result in the oxidation of cysteine 43 in the IGFBP-2 structure, increasing its bioavailability and enhancement of self-renewal—measured via increased neurosphere formation [143]. They additionally uncovered that the secretory pathway of IGFBP-2 may be redox-dependent, such that addition of hydrogen peroxide to NSC cultures in vitro resulted in greater detectable levels in the CM [143]. Therefore, the amount of ROS present in the microenvironment may be vital for the ability for IGFBP-2 to influence the balance between self-renewal and differentiation, and as such, studying the influence of IGFBP-2 under hypoxic conditions would be interesting. Lastly, analysis of IGFBP-2 binding to IGF-I and IGF-II revealed no redox dependent changes, and therefore the role of IGFBP-2 in regulating NSC maintenance and fates in this study was found to be independent of these ligands.

## 4. Glioblastoma: Identifying the Cell of Origin

The clinical presentation of glioblastoma often takes place at advanced stages of tumour development due to non-specific symptoms such as headaches, fatigue, nausea and vomiting [146,147]. Therefore, the time to diagnosis from symptom onset is <6 months in 85% of patients [148]. Previously, secondary glioblastoma (*isocitrate dehydrogenase* (*IDH*) mutant) was used to describe a tumour that had progressed from a lower-grade neoplasm [149,150]. However, the reclassification of glioblastoma in 2021 by the World Health Organisation (WHO) as a separate entity (only including *IDH* wildtype tumours) has highlighted the de novo nature of the tumour, and streamlined approaches to identify the tumour-initiating cell [2,151]. It is hoped that identifying the mechanisms that regulate tumour initiation, propagation and treatment evasion could lead to approaches to prevent and treat glioblastoma.

Leukaemia cells capable of tumour generation and mouse lethality were first observed in 1937 followed by the identification of leukaemia cells with enhanced self-renewal, believed to arise from malignant transformation of a primitive hemopoietic stem cell [152,153]. Subsequent revelations that cancer stem cells (CSCs) could be identified and isolated from breast, prostate, colorectal, pancreatic and brain cancer took place in the early 2000s [154,155,156,157,158,159,160,161]. In glioblastoma, these cells are now widely referred to as GSCs.

Despite this, the cellular origin of glioblastoma is still regarded as an open question. Rapid evolution and dynamics driving vast heterogeneity can quickly mask the original genetic features of the tumour. Therefore, there are several candidate untransformed cell types with the potential to propagate a tumour following oncogenic transformation, including NSCs, OPCs and astrocytes [162,163]. It is worth noting that studies such as those by Bachoo et al. (2002) showed that NSC and astrocytic transformation via deletion of the Ink4a/Arf locus (that encodes Retinoblastoma (RB) and p53), and epidermal growth factor receptor (EGFR) overexpression yielded tumours phenotypically similar to high-grade gliomas in vivo [162]. However, it remains to be determined whether the genetic manipulation of mature cells in vitro is reminiscent of the events that take place during glioblastoma development [164]. The delineation of early disease markers for detection or disease prevention may rely on identification of the origin cell.

### Evidence for Neural Stem Cells as the Glioblastoma Cell of Origin

Transcriptional and genetic evidence suggests that malignant alterations of NSCs could lead to the growth of gliomas. It is increasingly apparent that subsets of cells isolated from glioblastoma are genetically and transcriptionally regulated by cues associated with immature and progenitor cell populations [16,160,165]. Genome-wide CRISPR-Cas9 screening of 10 patient-derived GSCs and two NSCs by Macleod et al. (2019) revealed that despite the diverse molecular alterations of glioblastoma, a core gene set governs both GSC and NSC maintenance and growth including SOX genes, oligodendrocyte transcription factor 2 (OLIG2) and SALL1 [166,167].

Several studies have shown that combinations of TSG mutations/allele deletions (*tumour protein p53* (*TP53*), *Neurofibromatosis type 1* (*NF1*), *PTEN* and *RB1*) in the NSCs of mouse models lead to the development of high-grade glioma [168,169,170,171]. Follow-up studies by Llaguno et al. (2019) confirmed that *NF1*, *TP53* and *PTEN* allele deletions in early-stage progenitors were most likely to lead to glioblastoma development [172]. By inducing the same gene allele deletions in neuroblasts and terminally differentiated neurones reminiscent of adult neurogenesis, they found that early-stage cells (NPCs) were more likely to undergo malignant transformation than neuroblasts and differentiated neurones, with OPCs least resistant overall [172]. Indeed, it has been suggested that OLIG2-positive OPCs, which make up the largest group of dividing cells in the adult brain, are a major progenitor for glioma and medulloblastoma formation [163,171,172,173,174,175,176]. Moreover, OPCs are the major cell type produced by SVZ-NSCs, and markers (OLIG1/2, platelet-derived growth factor receptor (PDGFRα) and NG2) have been repeatedly recorded histologically in human gliomas and those induced in mouse models [173,177,178,179]. Moreover, by carrying out lineage tracing from an NSC with *TP53* and *NF1* deletions, Liu et al. (2011) showed that despite retaining neural and glial lineage formation capacity, only hyperproliferative-derived OPCs resulted in tumour formation [163].

**Figure 4:**
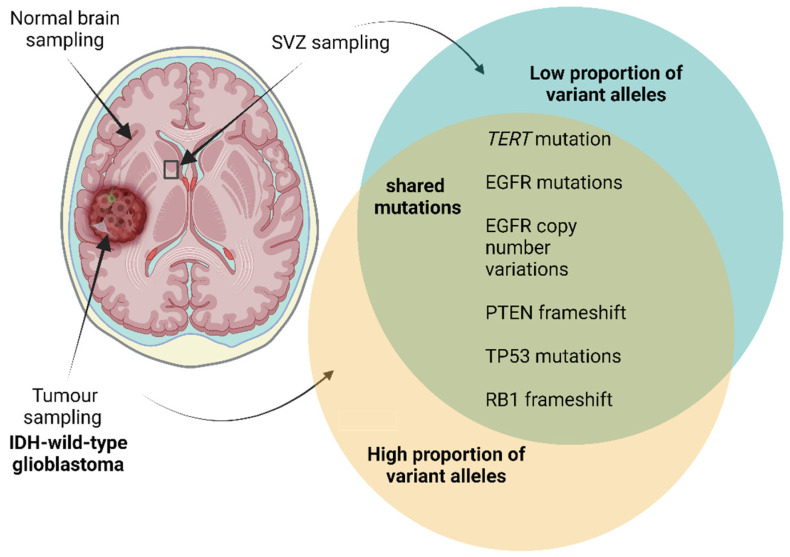
Schematic representing the findings in isocitrate dehydrogenase (*IDH*)-wildtype temporally located glioblastomas from Lee et al. (2018) [180]. Samples taken from SVZ free from tumours and the tumour itself were subject to whole-exome sequencing. Matching mutations between the two sites were found including single nucleotide variants in epidermal growth factor receptor (*EGFR*), tumour protein 53 (*TP53*) and telomerase reverse transcriptase (*TERT*). Insertions or deletions in phosphatase and tensin homolog (*PTEN*) and retinoblastoma protein (*RB1*) were also detected. The proportion of variant alleles or the variant allele frequency was much lower in SVZ samples than in tumour tissue (*TERT* C228T mutation SVZ: 1–22%, tumour: 22–52%). The figure is a visualisation of the results presented in [180]. Created with BioRender.com.

In 2018, Lee et al. published a study showing that 56.3% of NSCs isolated from the SVZs of 26 patients with temporal lobe-located *IDH*-wildtype glioblastoma exhibited low-level matching driver mutations to the tumour (Figure 4) [180]. Notably, *TERT* promoter mutations were detected at much higher rates in NSCs from patients with *IDH*-wildtype glioblastoma than controls. Furthermore, the induction of clinically relevant driver mutations—*TP53*, *PTEN* and *EGFR*—in SVZ cells led to the development of brain tumours that were analogous to high-grade proliferative gliomas in 9/10 mouse models [2,180]. Deep whole exome sequencing of SVZ samples away from the tumour mass is the most convincing evidence to date that the SVZ-NSC population in the adult human brain is a source for glioblastoma development [180]. Furthermore, Mendelian randomisation studies suggest that *TERT* promoter mutations may be an early indicator of the ability of NSCs to avoid replicative senescence, instead prolonging self-renewal capacity and the likelihood of further mutational acquisition [181,182]. It is worth noting that SGZ-NSCs appear to be less susceptible to malignant transformation—reviewed by Fontan-Lozano et al. (2020) [183].

Glioblastomas are closely associated with the SVZ in 50–60% of cases and manifest a more aggressive disease [97,184,185,186]. Moreover, these tumours are associated with a shorter survival post-resection [97,185,187,188,189,190]. Brain swelling and enhanced CSF brain volume confer a reduced OS of glioblastoma patients [191]. SVZ-directed radiotherapy post-resection results in significant increases in overall survival (OS) and progression-free survival (PFS) [192]. Furthermore, a retrospective review of glioblastoma tumours from the initial diagnostic MRI and resulting clinical outcome data, often referred to a “Lim classification”, has prompted subdivision into type (I–IV) tumours based upon the degree of SVZ involvement [185,189]. Those patients with SVZ-associated disease display more rapid tumour recurrence and a decreased OS and PFS [185,189,193]. In response to these observations, a call for intensive research into “SVZ-associated glioblastoma” and translation into clinical decision making was made in 2016 by Smith et al. [97].

**Figure 5:**
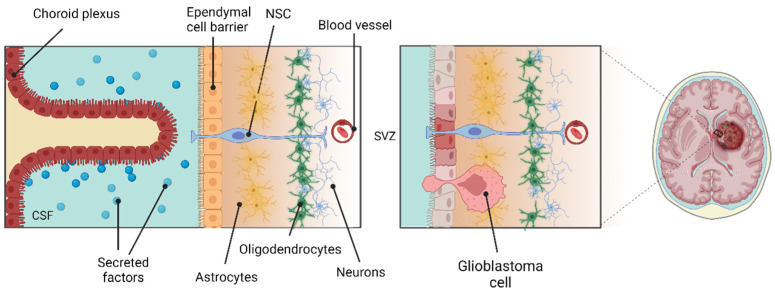
Schematic showing disruption of the ependymal lateral ventricle wall by glioblastoma tumour cells. Reports by Norton et al. (2022) described the ability for patient-derived glioblastoma cells to contact CSF via invasion of the lateral ventricular lumen [186]. As a result, metabolic homeostasis is lost and ependymal lipid biosynthesis is disrupted [199,200]. Ependymal functional cilia for CSF circulation, neuroblast migration to the olfactory bulb and protection from fluid build-up (hydrocephalus) become shortened and dysfunctional [125,186,201,202,203]. It is proposed that CSF and protein contents could infiltrate through disruption of the ependymal monolayer into the tumour [186]. This is one proposed reason for lateral ventricle proximal glioblastomas exhibiting enhanced malignancy [186]. Adapted from [126,127,186]. Created with BioRender.com.

The repeated isolation of well-established CSF-derived morphogens and growth factors including FGF2, EGF, Shh, retinoic acid and Wnt5a in combination with less-well characterised components such as IGFBP-2 and IGF-II underscores the importance the ChP secreted niche [194,195,196,197,198]. It is not unreasonable to suggest that alterations to the CSF milieu as a result of brain malignancy or injury may be a major source of inflammatory or tumorigenic signals. Indeed, in 2022, it was shown that gliomas in close proximity to the lateral ventricle cause ependymal barrier disruption and increased tumour–CSF contact [186] (Figure 5).

## 5. IGFBP-2, IGF-II and Glioblastoma

### 5.1. IGFBP-2 and Glioblastoma

Early microarray analysis of glioblastoma by Rickman et al. (2001) revealed 360 unique genes when compared with pilocytic astrocytoma including *IGFBP2*, *murine double minute 2* (*MDM2*), *CD44* and *cyclin-dependent kinase 4* (*CDK4*) [204]. In the early 2000s, widespread gene expression profiling to assist histological tumour evaluation potentiated studies evaluating gene predictors of glioma malignancy and patient survival [205,206,207,208]. In 2007, the combined absence of IGFBP-2 and IQ motif containing GTPase-activating protein 1 (IQGAP1) protein expression in glioblastoma patient tissue was shown to correlate with longer survival (>3 years) in studies by McDonald et al. (2007) [209]. Furthermore, in 2010, Colman et al. reviewed transcriptional findings from four independently published glioblastoma datasets [210]. This, in combination with analysis of glioblastoma formalin-fixed paraffin-embedded tissue samples identified a nine-gene signature including IGFBP-2, chitinase-3-like protein 1 (CHI3L1)/YKL-40, galectin 3 (LGALS3) and OLIG2 that was predictive of worse clinical outcomes [210]. Unsurprisingly, poorly differentiated tumours expressing nestin and CD133 were also associated with the nine-gene signature and worse responses to current treatment regimens [210]. Further molecular profiling of 1122 diffuse glioma samples by Ceccarelli and colleagues in 2016 revealed IGFBP-2 protein elevation in glioblastoma with EGFR, P-EGFR and P-Akt, when compared with lower-grade gliomas (2016 CNS WHO grades II and III) [211]. A prognostic relationship between high-grade glioblastoma, *IGFBP2* expression and the incidence of *TERT* promoter mutation was further described by Yuan et al. (2019) [9]. The most prevalent *TERT* promoter mutation recorded in this study was C228T—previously suggested as an early event in the transformation of NSCs [9,180].

Within the last 5 years, efforts to stratify and optimise glioblastoma patients for predicted responses to therapy by Yin, Prasad, Yu and colleagues independently identified IGFBP-2 in gene signatures (with under 20 genes) associated with worse outcomes [212,213,214]. The identification of such gene signatures with predictive and prognostic utility is hoped to drive the development of novel therapeutic options for patient subsets or enable the gene signatures to act as therapeutic targets themselves. Furthermore, an assessment of IGFBP expression levels across 19 different cancer types deposited by The Cancer Genome Atlas in 2023 found that *IGFBP2* mRNA levels were significantly associated with the prognosis of low-grade glioma (LGG) and glioblastoma (*p*-values: 8.3 × 10^−33^ and 1.2 × 10^−3^, respectively) (hazard ratios (95% confidence interval): 1.55 (1.44,1.68) and 1.24 (1.08, 1.40), respectively) [215].

Many mechanistic studies of IGFBP-2 thus far have been carried out in immortalised glioblastoma cell lines such as U251, U87 and T98G due to their ease of use and relatively low cost to maintain. However, the addition of animal serum into culture media and in vitro passaging facilitate the acquisition of genetic and epigenetic alterations over time, unlikely to maintain the gene expression profiles present in the glioblastoma from which they were derived [165]. Moreover, it is important to note that despite studies showing the low expression of IGFs from these glioblastoma cells themselves, the inclusion of animal serum introduces IGFs and other growth factors, confounding the ability to determine whether the effects of IGFBP-2 in these studies are IGF-independent [216].

In 2014, Han et al. showed that the exogenous delivery of IGFBP-2 to U87, SU3 and U251 cells promoted invasion and proliferation, whilst endogenous upregulation was specific to increased invasive potential [14]. The proliferative and invasive effects upon recombinant human IGFBP-2 dosing were attributed to integrin β1, downstream extracellular signal-related kinase (ERK) phosphorylation and nuclear translocation of IGFBP-2 [14]. The study additionally showed that exogenous IGFBP-2 integrinβ1/ERK pathway signalling resulted in abrogation of temozolomide (TMZ) activity, which was not replicated upon endogenous overexpression/knockdown. In another study, exogenous activation of integrin α5β1, downstream focal adhesion kinase (FAK)/ERK and JUN NH_2_ terminal kinase (JNK) phosphorylation, and subsequent transcriptional upregulation of VE cadherin (CD144) and MMP2 in U87, and primary glioblastoma cells was shown by Liu et al. [11]. In agreement with studies by Han et al. (2014), *IGFBP2* knockdown in high-*IGFBP2*-expressing U251 cells decreased IGFBP-2 secretion levels and cellular migratory capacity, but did not affect proliferation [11,14]. Similarly, the interaction between IGFBP-2 and integrin α5β1 in SNB19 glioblastoma cells overexpressing IGFBP-2 was shown to activate JNK with downstream cell migration enhancement [12]. Ingenuity pathway analysis of the Repository for Molecular Brain Neoplasia Data (Rembrandt) by Holmes et al. also showed that cellular migration and invasion pathways including integrin linked kinase (ILK) and nuclear factor kappa-light-chain enhancer of activated B cells (NF-ΚB) are associated with IGFBP-2 [10]. In vitro knockdown studies showed that ILK and NF-ΚB activation was downstream of IGFBP-2-integrin α5β1 binding and resulted in the transcription of invasion-related genes [10]. Additionally, in 2016, following delineation in breast cancer, Patil et al. showed that the association between the arginine–glycine–aspartate (RGD) domain of IGFBP-2 and integrins could lead to the stabilisation of nuclear β-catenin, contributing to tumour growth [217,218]. An RGD containing the C-terminal but not N-terminal fragment of IGFBP-2 led to FAK activation and inactivation of glycogen synthase kinase (GSK3β)—required for β-catenin phosphorylation and degradation via the proteosome [217]. Upstream IGFBP-2 activation of Wnt/β-catenin signalling has more recently been shown to induce EMT in hepatocellular carcinoma [219]. Nuclear accumulation of β-catenin in glioblastoma cells led to the upregulation of transcriptional targets including Oct-4, MMP-2, Nanog and c-Myc [217].

Despite a wealth of research that indicates that IGFBP-2 exhibits oncogenic properties within glioblastoma, the specific effect of IGFBP-2 on the stem cell compartment (NSCs and GSCs) is sparse. Quantitative polymerase chain reaction (qPCR) analysis of GSCs, normal brain tissue and differentiated glioblastoma cell lines by Hsieh et al. (2010) showed that *IGFBP2* was overexpressed in GSCs independently of *IGFBP5*, *IGF1* and *IGF1R* [220]. Moreover, parallel secretome profiling between serum-cultured GSCs and their stem-like equivalents was recently investigated by Robilliard et al. (2022) [221]. The authors suggest how imperative different cellular states may be in modulating the surrounding immune microenvironment. High expression of immune modulatory genes including *IGFBP2* was detected in serum-cultured cells/GSCs via NanoString analysis [221] Moreover, 5–50-fold higher levels of secreted proteins were detected from serum-derived cultures. However, simultaneous proteome profiling of cells cultured in both serum and serum-free conditions detected an elevated IGFBP-2 abundance in GSC cultures. Since *IGFBP2* and *VEGFA* mRNA were significantly associated with elevated macrophages in the dormant state (M0) and decreased anti-inflammatory state (M2), the authors suggest that elevated IGFBP-2 secretion from GSCs could be an important factor for shaping the glioblastoma immune microenvironment. Finally, it was proposed that IGFBP-2 may serve to maintain the stemness of GSCs via autocrine signalling [221].

Experimental knockdown of *IGFBP2* in GSCs by Hsieh et al. (2010) decreased the expression of *SOX2*, *NES*, *BM1* and *CD133*, indicating a role of IGFBP-2 as part of the GSC transcriptional network [220]. The link between SOX2 abundance and IGFBP-2 was further elucidated in studies by Berezovsky et al. (2014) [222]. The group showed that patient-derived GSCs exposed to serum retained the potential to differentiate via SOX2. However, this was abolished when *SOX2* was silenced [222]. Moreover, *SOX2* knockdown mediated differential expressions of genes involved with embryogenic stem cell renewal, cytokine signalling and malignancy including a 6-fold downregulation of *IGFBP2* [222]. In medulloblastoma—a childhood brain tumour—IGFBP-2 is instrumental for the SOX2-driven Shh subtype. In this study, IGFBP-2 was shown to mediate primary patient cell proliferation and migration through a signal transducer and activator of transcription 3 (STAT3)-mediated pathway [223]. In addition, using a genetic mouse model of medulloblastoma, Vanner et al. (2014) showed that Shh subgroup cells could maintain quiescent states during anti-mitotic chemotherapy through SOX2 positivity [224]. Therefore, there may be a functional relationship between IGFBP-2 and SOX2 that has not yet been explored in specific subtypes of CSCs.

### 5.2. IGF-II and Glioblastoma

In vitro studies with immortalised glioblastoma cell lines including U87, U251, U373 and T98G show low expression of the *IGFRs*, and in many cases lack cellular IGF-I or -II [225,226,227]. As follows, several studies in these cell lines found that IGF-I or -II treatment caused little mitogenic effect in vitro and in vivo [225,226]. ISH or IHC of astrocytoma and glioblastoma tissue has detected the *IGF1R*, *IGF1* and *IGF2*, although overall *IGF2* seems much less prevalent (5/88 positive glioblastomas, Soroceanu et al. (2007); 29/50 primary and secondary glioblastomas, Suvasini et al. (2011)) [228,229,230,231,232]. Early studies comparing normal brain and mixed glioma specimens largely indicated elevated tumour *IGF1* and *IGF2* expression and protein abundance; however, these reports suffered from ambiguity due to small sample sizes and differing detection methods [228,229,233,234]. Larger-scale comparison of low/high-grade astrocytomas, glioblastomas and normal human brain tissue showed no significant differences in *IGF* expression [232,235,236]. Moreover, 78% of 218 glioblastoma samples studied by Maris et al. (2015) showed no positivity for IGF-II when analysed by IHC [237]. Interestingly, however, of the glioblastomas with positivity, IGF-II has been mostly identified in peri necrotic areas, suggestive of a hypoxic relationship in these tumours [237,238].

It is worth noting that a small subset of glioblastomas with elevated *IGF2* expression may be prognostically relevant. Further investigation of this subgroup by Soroceanu et al. (2007) demonstrated that 16/121 glioblastomas in their study displayed enhanced *IGF2* expression and were associated with a lack of EGFR modifications, loss of *PTEN*, elevated levels of the IGF-1R, PI3K/P-Akt proliferation and poorer patient survival [231,235]. Indeed, in relation to the findings by Mu et al. (2014), it is possible that IGF-II upregulation is activated in a subset of glioblastomas by insulin-like growth factor 2 mRNA-binding protein 2 (IMP2) [239]. This study showed that IMP2 can regulate IGF-II activity, to promote downstream PI3K/Akt signalling [239].

As referred to previously, there have been no experimental approaches to study the effects of IGF-II on the patient-derived GSC population. This, however, would be interesting since IGF-II activity has been shown to substitute for EGF and can cooperate with FGF to maintain stem cell potency and survival [240]. Furthermore, IGF-II appears to support the growth of neurospheres derived from glioma cell lines in vitro via the activation of IGF-1R and downstream PI3K-Akt signalling [231].

## 6. Discussion

Glioblastoma is a poorly differentiated tumour, strongly aligned with an NSC origin [180,241]. Studies to investigate the cell of origin are essential for understanding the biological basis of and clinical implications for glioblastoma [242]. GSC populations create a moving target, associated with treatment evasion and the inhibition of long-term remission [243]. Standard treatment regimens including radiotherapy and TMZ chemotherapy require the presence of actively cycling cells. Many of the regulatory cues required for the quiescent/activated states of NSCs are largely understudied but could be essential for cellular ‘priming’ to cycling and a targetable state [244,245]. Therefore, the unique biochemistry of the brain, glial/neural cell biology and localised secretion could all be essential in the establishment of the heterogeneity for which glioblastoma is so well known.

Several studies have reported IGFBP-2 overexpression in glioma of increasing grade and aggression [246,247]. Many of these studies also present a strong prognostic role for IGFBP-2 [9,210]. In vitro studies including immortalised glioblastoma cell lines also reveal higher expression than normal brain samples under serum-cultured conditions, and several functions have been elucidated in other neoplasms [216]. Despite the inverse relationship between tumour IGFBP-2 abundance and survival, the mechanistic basis and functional consequences are poorly understood.

IGFBP-2 is highly expressed in the prenatal CNS [76,248]. It has been recorded at key regions of differentiation priming, morphogenesis and growth [249]. In particular, the notochord, which has the capacity to differentiate into the floor plate for further development of the nervous system of rats, coincides with high IGFBP-2 expression [250,251]. A high abundance of IGFBP-2 in proliferative NSCs in the mouse neural tube at E11.5 shows high correlation with and apical polarity with nestin and SOX2 [252]. The elevation of neuron-specific βIII-tubulin (TUJ1) and GFAP at E17.5 coincides with decreased IGFBP-2 [252]. Intricate expression patterns in mid-gestational rat embryos by Wood et al. (1992) showed that transitioning neuroblasts increased the neuronal intermediate filament protein alpha-internexin and lost IGFBP-2 [249,253]. *IGFBP2* knockdown studies also highlighted an increased capacity for differentiation of mouse NSCs [252]. The abundance in self-renewing populations is indicative of a role for IGFBP-2 in their maintenance prior to lineage commitment and differentiation induction. Furthermore, established roles in brain development have been supplemented by emerging functions for higher-order brain functioning into adulthood [254,255,256].

Although IGF-II does not seem to be overexpressed in glioblastoma cells, the study of 88 glioblastomas by Soroceanu et al. (2007) suggest that there could be a subset of patients with high *IGF2* expression [231]. Moreover, as mentioned previously, the measurement of high-molecular-weight variants of IGF-II requires further study. Notably, the secretion of high-molecular-weight IGF-II has been detected in the serum of cancer patients, with a direct link to non-islet tumour cell hypoglycaemia—reviewed by Van Doorn et al. (2020) [257]. Whether these variants are similarly enriched in glioblastoma remains unconfirmed. Similarly, given their preserved receptor activation capacity, but potentially altered affinity for IGF-binding proteins, including IGFBP-2, it is plausible that high-molecular-weight IGF-II may differentially modulate IGF bioavailability and receptor activation in the glioblastoma microenvironment [258,259]. Indeed, the detection of a 9 kDa variant in human CSF reported by Haselbacher et al. (1982) warrants further investigation in this context [68].

Drawing from studies in NSPCs, the interaction between IGF-II and IR-A appears to be important for cell clonogenicity and stemness [139]. However, despite evidence for the overexpression of IR-A in aggressive cancers, there is a lack of study on glioblastoma [260,261,262]. Again, despite a lack of research in patient-derived GSCs, there is evidence to suggest that IR-A may enhance cancer stemness in hepatocellular carcinoma (HCC) [263]. Of note, the use of IGF-II neutralising antibodies holds promise in slowing the growth of mouse HCC xenografts in mice [264].

In the adult human brain, IGFBP-2 is locally produced and too large to cross the BBB into systemic circulation and vice versa [76]. Postnatal retention of *IGFBP2* expression by ChP epithelia and hippocampus localises IGFBP-2 to areas of adult NSCs [114,122]. Fluorescent labelling of mouse SVZ and SGZ regions postnatally indicated that *IGFBP2* was restricted to cells with nestin positivity [252]. Similarly, *IGF2* is expressed by ChP and can be detected at higher levels in the CSF of glioblastoma patients [120]. With a molecular weight of 6765 Daltons, IGF-II can cross the BBB and could also be delivered to the tumour via the vasculature [78]. Modern secretome analysis is hoped to expand our understanding of cellular crosstalk and signal transduction in disease [265]. With many of the available methods requiring pre-selected antibody arrays, the development of unbiased methods such as LC-MS/MS may provide more comprehensive characterisation of secreted factors [114].

The modulation of IGFs is a well-established role for IGFBP-2 with downstream mitogenic and systemic effects including metabolism [266]. Therefore, the extracellular role of IGFBP-2 and IGF-II in close proximity to SVZ or SGZ NSCs could be described by canonical IGF signalling. However, this does not explain the overexpression and prognostic effect of IGFBP-2 in glioblastoma studies. Therefore, independent functioning also seems likely, relying on the additional functional motifs present within the IGFBP-2 structure [77]. On the other hand, the role of IGF-II still requires elucidation, and its expression may be prognostically relevant in a subset of patients. Lastly, the proximity of SVZ NSCs to the CSF raises the possibility that IGFBP-2, IGF-II and other understudied factors here could be involved in CNS disease [122].

## 7. Conclusions

To conclude, the study of NSCs, their states and how these are regulated may be imperative to our understanding of glioblastoma initiation. The expressions of *IGFBP2* and *IGF2* are retained in the adult human brain, most notably in the ChP and derived CSF—highlighting them both as interesting constituents of the adult SVZ NSC niche [122,267]. Moreover, IGFBP-2 has been detected in the CM of NSCs in vitro [110,114]. Studying how these factors regulate NSCs and their progenitors may provide clues as to how they modulate GSCs. Indeed, evidence is suggestive of the role of IGFBP-2 in NSC and GSC maintenance [220,252]. Although not well defined, the expression of *IGF2* in the adult ChP and CSF, combined with signalling though IR-A, supports a rationale for further investigation in the context of GSC maintenance. Further study should focus on IGFBP-2 and IGF-II as secreted factors and their functions, both dependent and independent of each other.

## Abbreviations

The following abbreviations are used in this manuscript:

APC	Astrocyte progenitor cell
APOE	Apolipoprotein E
BBB	Blood–brain barrier
BMP	Bone morphogenic protein
CDK4	Cyclin-dependent kinase 4
CHI3L1	Chitinase-3-like protein 1
ChP	Choroid plexus
CM	Conditioned media
CNS	Central nervous system
CSC	Cancer stem cell
CSF	Cerebral spinal fluid
ECM	Extracellular matrix
EGF	Epidermal growth factor
EGFR	Epidermal growth factor receptor
ERK	Extracellular signal-related kinase
ESC	Embryonic stem cell
FABP7	Fatty acid binding protein 7
FAK	Focal adhesion kinase
FBS	Foetal bovine serum
FGF	Fibroblast growth factor
GFAP	Glial fibrillary acidic protein
GSC	Glioma stem cell
IDH	Isocitrate dehydrogenase
IGF	Insulin-like growth factor
IGF-1R	Insulin-like growth factor I receptor
IGF-2R	Insulin-like growth factor II receptor
IGFBP	Insulin-like growth factor binding protein
IHC	Immunohistochemistry
ILK	Integrin linked kinase
IQGAP1	IQ motif containing GTPase-activating protein 1
IR	Insulin receptor
IRS	Insulin receptor substrate
ISH	In situ hybridisation
JNK	JUN NH_2_ terminal kinase
LC-MS/MS	Liquid chromatography with tandem mass spectrometry
LGALS3	Galectin 3
M6P	Mannose-6-phosphate
MAPK	Mitogen-activated protein kinase
MDM2	Mouse double minute 2 homolog
MEK	Mitogen-activated protein kinase
MMP	Matrix metalloproteinase
mRNA	Messenger ribonucleic acid
NF1	Neurofibromatosis type 1
NPC	Neural precursor cell
NSC	Neural stem cell
NSPC	Neural stem/progenitor cell
OLIG2	Oligodendrocyte transcription factor 2
OPC	Oligodendrocyte precursor cell
OS	Overall survival
PDGFR	Platelet-derived growth factor receptor
PFS	Progression free survival
PI3K	Phosphatidylinositol-3-kinase
PTEN	Phosphatase and tensin homolog
qPCR	Quantitative polymerase chain reaction
RB	Retinoblastoma protein
Rembrandt	Repository for Molecular Brain Neoplasia Data
RGC	Radial glial cell
RGD	Arginine–glycine–aspartate
RNA	Ribonucleic acid
ROS	Reactive oxygen species
Shh	Sonic hedgehog
SOX2	Sex determining region Y-box 2
STAT3	Signal transducer and activator of transcription 3
SVZ	Subventricular zone
SGZ	Subgranular zone
TERT	Telomerase reverse transcriptase
TGF	Transforming growth factor
TIMP1	Tissue inhibitor matrix metalloproteinase 1
TMZ	Temozolomide
TP53	Tumour protein p53
VEGF	Vascular endothelial growth factor
WHO	World Health Organisation
